# Analysis of Crack Image Recognition Characteristics in Concrete Structures Depending on the Illumination and Image Acquisition Distance through Outdoor Experiments

**DOI:** 10.3390/s16101646

**Published:** 2016-10-06

**Authors:** Hyun-Woo Cho, Hyuk-Jin Yoon, Jae-Chan Yoon

**Affiliations:** 1 Robotics and Virtual Engineering, University of Science and Technology, Uiwang-si 16105, Korea; hwcho@krri.re.kr; 2 Korea Railroad Research Institute, University of Science and Technology, Uiwang-si 16105, Korea; 3 Department of Civil Engineering, Han-Yang University, Seoul 04763, Korea; jcyoon@krri.re.kr

**Keywords:** concrete crack, illumination, shooting distance, MTF, image processing

## Abstract

The effects of illumination and shooting distance on crack image recognition were investigated by examining cracks in images taken with a camera. In order to examine the effects, images of cracks in a concrete structure taken while varying the illumination and shooting distance in an outdoor environment were analyzed. The images were acquired at a daytime illumination of 52,000 lx and a night illumination of 13 lx. The crack specimen images produced for the experiment were taken by increasing the shooting distance from 5 m to 100 m in each illumination. On the basis of the analysis on the modulation transfer function (MTF) and contrast sensitivity of the crack images, the effects of illumination and shooting distance on the sharpness of the crack images were investigated. The minimum crack widths that can be identified under each illumination were analyzed using MTF10 and Weber contrast 0.1, respectively. It was found that as the shooting distance increases, the effects of illumination on crack recognition become greater.

## 1. Introduction

Even now, when the forms of structures are ever changing, the number of concrete structures is increasing. As cracks occur in concrete structures and performance degradation appears, disasters resulting from collapse continue to occur, and the number of buildings that require repairs is also increasing. Cracks in concrete structures are often caused by problems in design and construction, sudden application of loads exceeding the design load, and repetitive loads applied over a long period of time [1]. If loads are applied to structures in which micro-cracks have occurred, cracks grow from the micro-cracks, and surface cracks during construction often lead to structural cracks [2]. The current safety inspection of structures poses disadvantages in that a lot of time and manpower are required due to the reliance on visual inspection by experts. Research on automatic crack recognition technology to combat these disadvantages using an image processing technique has been receiving attention. The crack image recognition technique can be used to monitor structures in a faster measurement cycle by reducing the required manpower and time, thereby enabling a quick response when serious defects occur in the structures [3].

Abdel-Qader et al. [3] extracted cracks from bridge images using fast Haar transform (FHT), fast Fourier transform, Sobel, and Canny, and provide a comparison of four crack detection techniques. Yamaguchi and Hashimoto [4] claimed that computation time is important in applying an image-based system to a practical application and contributed to the enhancement of image processing speed by proposing a percolation-based algorithm to calculate the circularity of an object during image processing and skip circular objects. Zhang et al. [5] proposed a method to extract crack images from noisy concrete surface images by using a distance histogram-based shape descriptor. Crack measurement using images has been studied mainly with respect to a variety of structures. Sinha [6] presented a method to extract cracks from buried concrete pipes, and Ho [7] presented a method for measuring the cable surface cracks of cable-stayed bridges. Wu [8] proposed a method for the accurate measurement of cracks in concrete road pavement. Li et al. and Zou et al. proposed the FoSA and CrackTree methods for crack detection from pavement images, respectively [9,10].

As such, crack measurement using images has been widely studied. However, in the previous studies, the cracks were extracted in a controlled environment. With nuclear power plants or hydroelectric dams, it is difficult to shoot close-up images, so the image shooting distance becomes large in these cases. In addition, changes in illumination occur according to the weather and time in an outdoor environment, and this effect should be considered when using crack recognition techniques. Meanwhile, if the shooting distance increases, the spatial resolution is reduced, and thus the contrast of the crack is lowered. In this case, the change of illuminance causes the contrast of the crack and concrete background to change. In the outdoor environment, the shooting distance and illuminance cannot be controlled, and these changes affect the crack extraction process using thresholding and boundary detection used in the previous studies, thereby having a significant effect on the crack extraction performance.

Jahanshahi and Masri [11] suggested a technique that enables crack detection and quantification at any focal length, shooting distance, and resolution by adding the use of depth perception to the crack image recognition technique in an outdoor environment. Jahanshahi et al. [12] analyzed printed crack images that had crack widths ranging from 0.4 to 2.0 mm by changing the shooting distance from 725 mm to 1760 mm, and determined that as the number of pixels that represent the cracks decreases, the accuracy of the crack width measurement is reduced, and a reduction in shooting distance, increase in focal length, and enhancement of resolution are needed to increase the accuracy of the crack measurement. Li et al. [13] propose a technique that can measure cracks in a bridge from a long distance. As the measurement distance increased, the crack measurement error became larger, while the light source and ISO did not have a significant effect. Wada and Kono [14] measured cracks in a hydroelectric dam with a size of 0.2 mm at a distance of 120 m using an 80–400 mm telephoto lens and a 12.6 Megapixel sensor. Jahanshahi [11,12] and Li et al. [13] concluded from their analysis that an increase in shooting distance leads to a reduction of the spatial resolution of the image, and the decrease in the number of pixels that represent the cracks lowers the accuracy of the crack measurement. Li et al. [13] analyzed the effect of illumination on the accuracy of crack measurement by varying the ISO values and the presence of a light source. In previous studies, the crack measurement error of the crack measurement algorithm developed was verified by varying the shooting distance and the presence of a light source with the acknowledgment that the light source and shooting distance have an effect on the accuracy of the crack measurement [11,12,13]. However, though the measurements showed that changes in the light source and shooting distance caused crack measurement error, the effects on image recognition were not quantitatively analyzed.

In this study, a quantitative analysis of the effects of image acquisition conditions on the surface crack recognition in the specimen image of the concrete structure was conducted through an outdoor experiment. A concrete specimen with openings at regular intervals was fabricated, and the specimen was made to be similar to the bar target so as to facilitate modulation transfer function (MTF) analysis. Images of the concrete crack specimen were taken while increasing the shooting distance according to changes in the illumination occurring during the daytime. Through the inspection of cracks using the images taken during the daytime, the range of crack widths that can be recognized depending on the shooting distance and changing illumination were determined through experimental methods. For the image analysis, image evaluation methods such as edge response analysis and modulation transfer function (MTF) were used, and the effects on human visual perception were objectively analyzed using contrast sensitivity and Munsell value.

## 2. Crack Image Analysis Techniques

The ability to recognize cracks in an acquired image is related to the shooting distance and spatial resolution of the camera. The spatial resolution represents the ability of the image acquisition system that can express fine details in the image. The spatial resolution can be calculated using the resolution of the sensor and the focal length of the lens, through which the measurable crack width can be estimated. The spatial resolution is the spatial extent that one pixel of the image occupies in the ground, and the theoretical spatial resolution can be obtained using the geometric relationship between the variables of the camera as shown in Equation (1):

(1) $$x_{GSD}=\frac{x_{\mathrm{image}}\times D_{w}}{f}$$

(2) $$x_{\mathrm{image}}=\;\frac{S_{s}}{S_{R}}$$

*x_GSD_* represents the distance of the ground that one pixel of the image has, *x*_image_ the size of pixel, *D_w_* the shooting distance, and *f* the focal length. *S_s_* in Equation (2) is the sensor size, and *S_R_* the resolution of the sensor.

However, in the case where the camera is applied to a real environment, a difference arises between the theoretically estimated spatial resolution and the actual resolution. For that reason, research to verify the spatial resolution of the camera in the actual environment has been conducted. Mraz et al. presented a method for verifying the performance of a digital imaging system used in a highway application [15]. They pointed out that the specification provided by the camera manufacturer has a low level of reliability, and the quality of the image cannot be accurately predicted when other optical systems such as lenses are connected to the camera, and also proposed a method to analyze the factors affecting the image quality by using an optical theory.

### 2.1. Crack Edge Response Analysis

**Figure 1:**
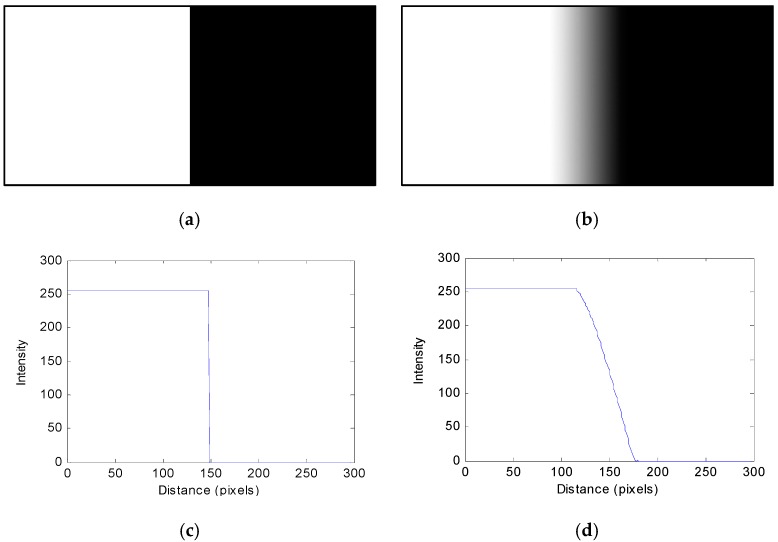
Edge analysis in the crack image: (**a**) object, (**b**) image, (**c**) ESF of object, (**d**) ESF of image.

Crack edge response analysis is a method for evaluating the sharpness of the boundary between the dark and bright areas in the acquired image. The image obtained by taking a picture of the actual object with a distinct boundary as shown in Figure 1a has blurred edge due to the blur phenomenon, as shown in Figure 1b [16].

**Figure 2:**
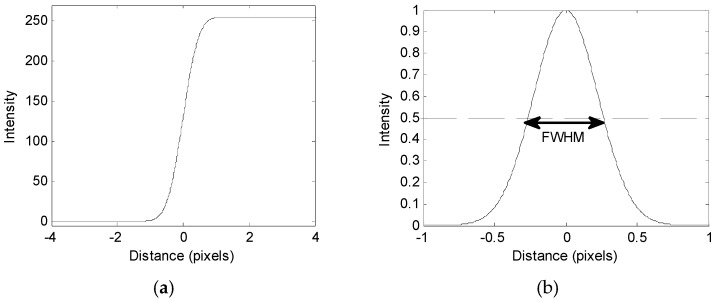
Edge response: (**a**) Fitted sigmoid function into the edge profile, and (**b**) first derivative of (**a**) with marked full width at half maximum.

The edge spread function (ESF) is the response of the system to an ideal edge [17]. The point spread function (PSF), which is the first-order partial differential function of ESF, is commonly used to indicate the quality of the imaging system. PSF represents the diffusion degree of the points in the image and is used to evaluate the sharpness of the image. Figure 2b shows PSF and full width of half maximum (FWHM). In a PSF function, the sharpness is evaluated based on the FWHM size that corresponds to the width at half of the maximum amplitude [16]; the better the sharpness, the smaller the FWHM.

### 2.2. Modulation Transfer Function Method

Modulation Transfer Function (MTF) is one of the techniques for evaluating the spatial resolution. The methods used to obtain MTF include an edge method and a sine wave method. The edge method is used to obtain LSF by differentiating the ESF extracted from the boundary between the bright and dark sides and calculating MTF through a Fourier transform.

In order to convert ESF into MTF, the LSF function should be calculated first, and LSF can be obtained by differentiating ESF as shown in Equation (3) [18]:

(3) $$\frac{d}{dx}\left\{ESF\left(x\right)\right\}=\frac{d}{dx}\int_{-\infty }^{x}LSF\left(x^{\prime }\right)dx^{\prime }=LSF\left(x\right)$$

The transform of LSF is obtained via the Fourier transform, and the optical transfer function (OTF) is calculated as shown in Equation (4):

(4) $$\mathrm{OTF}\left(\xi \right)=\;\int_{-\infty }^{\infty }LSF\left(x\right)\cdot e^{-j2\pi \xi x}dx$$

ξ is the frequency, where OTF (Optical transfer function) contains both the phase and amplitude information of the signal, which is converted to DFT as shown in Equation (5):

(5) $$\mathrm{OTF}\left(\xi \right)=\;\frac{1}{N}\overset{N-1}{\underset{n=0}{\sum }}LSF\left(n\right)\cdot e^{-\xi \left(2\pi \frac{n}{N}\right)}\;$$

(6) OTF(ξ)=A−iB

The DFT of Equation (4) can be represented by Euler’s formula as in Equation (6), where *A* and *B* are shown in Equations (7) and (8), and MTF can be obtained as the absolute value of OTF as shown in Equation (9):

(7) $$A=\frac{1}{N}\overset{N-1}{\underset{n=0}{\sum }}LSF\left(n\right)\cdot \cos\left(2\pi \xi \frac{n}{N}\right)$$

(8) $$B=\frac{1}{N}\overset{N-1}{\underset{n=0}{\sum }}LSF\left(n\right)\cdot \sin\left(2\pi \xi \frac{n}{N}\right)$$

(9) $$\mathrm{MTF}\left(\xi \right)=\left|OTF\left(\xi \right)\right|=\sqrt{A^{2}+B^{2}}$$

where *N* is the total number of data points and *n* is an integer.

Another MTF measuring method involves taking a picture of the bar target with a camera; the MTF value is calculated as the relative ratio of modulation (*M_i_*) of the image and modulation (*M_o_*) of the object, as shown in Equation (10) [15]:

(10) $$\mathrm{MTF}=\;\frac{M_{i}}{M_{o}}$$

First, the modulation value for the target region should be calculated. For reflecting targets, M_o_ is defined as shown in Equation (11) [15]:

(11) $$M_{o}=\frac{\left(R_{\max}-R_{\min}\right)}{\left(R_{\max}+R_{\min}\right)}$$

$R_{\max}$ and $R_{\min}$ are the maximum and minimum reflectance at a given uniformly illuminated background [15]. $M_{i}$ is defined as in Equation (12):

(12) $$M_{i}=\frac{\left(I_{\max}-I_{\min}\right)}{\left(I_{\max}+I_{\min}\right)}$$

$I_{\min}\;\mathrm{and}\;I_{max}\;$are the maximum and minimum intensity values of the image [15]. The actual image taken by the camera has lower resolution and contrast than the object, and the MTF value is a measure of the transformation of the spatial frequency entered in the process of creating the image.

### 2.3. Contrast Sensitivity Analysis

The contrast in the image that can be perceived by humans varies depending on the intensity of the luminance, and the width of the PSF cannot represent human perception. In order to quantify the ability to distinguish the contrast by considering the human color perception capability, studies on the contrast sensitivity have been conducted. The contrast sensitivity defines the threshold between visible and invisible. The contrast is the value obtained by quantifying the relative luminance of the background and the object, and is represented using Weber contrast (C_W_) and Michelson contrast (C_M_) methods [19]:

(13) $$C_{W}=\frac{L_{\max}-L_{\min}}{L_{\mathrm{Back}}}\;$$

(14) $$C_{M}=\frac{L_{\max}-L_{\min}}{L_{\max}+L_{\min}}$$

where $L_{\max}$ and $L_{\min}$ are the maximum luminance and minimum luminance, and $L_{\mathrm{Back}}$ is the luminance of the background.

**Figure 3:**
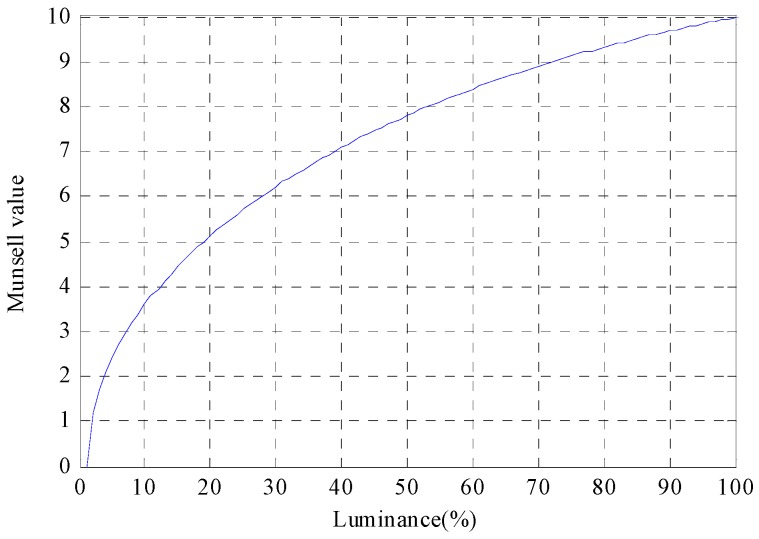
Munsell value vs. luminance.

The ability to distinguish the structure through the contrast in the image can also be analyzed through a Munsell value. The Munsell value is known as the lightness scale with perceptually uniform intervals. With respect to the relationship between luminance and Munsell value, as the luminance becomes higher, it is difficult to distinguish the difference in the luminance that can be perceived as shown in Figure 3 [20].

## 3. Experimental Apparatus and Method

### 3.1. Specimen Production

A specimen in which cracks are artificially created in a concrete structure was produced, and it was made to have 10 widths of cracks ranging from 2 mm to 20 mm at 2 mm intervals. The specimen was created by mixing mortar cement and water with a mixing ratio of 25 kg: 1.2 L, and the compressive strength was more than 10 MPa after curing for 28 days.

**Figure 4:**
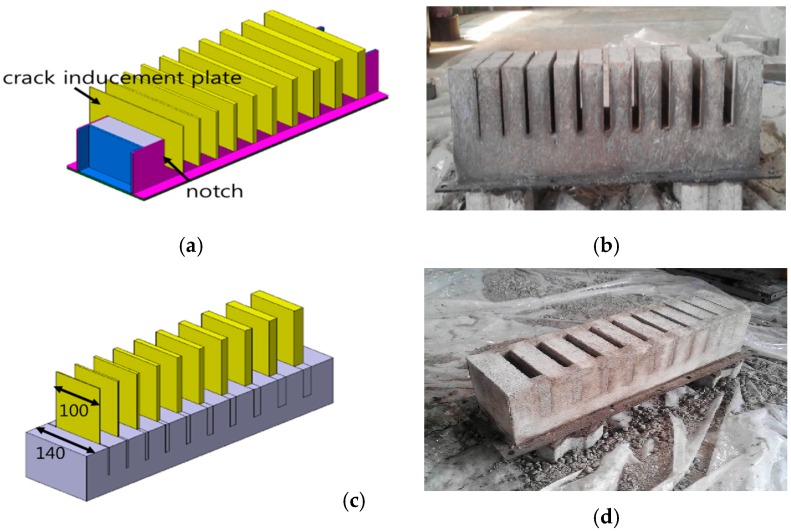
Crack specimen production method and crack specimen photo: (**a**) Crack generation technique using crack inducement plate; (**b**) Crack specimen generated by crack inducement plate; (**c**) Technique for side wall generation by removing the crack inducement plate after mortar cement placement; (**d**) Crack specimen in which the side wall is generated.

Figure 4 shows a method for crack specimen production and photos of the crack specimen. A mold for concrete placement was produced, and notches with the same thickness and depth as the cracks were made on both sides of the mold wall, after which a crack inducement plate (CIP) with the same thickness as the crack width to be generated was inserted into each respective notch. After the placement of mixed concrete in the mold, it was cured at room temperature. The CIPs were removed first after curing for about 40 h, and the two side walls of the mold were removed after curing for about eight days. Figure 4b shows the cracks generated by the crack inducement plates, with the generated crack widths showing an error of less than 0.2 mm. Since the two side walls are open, an operation to fill the side walls was performed as shown in Figure 4c. After CIPs of 100 mm in length—40 mm shorter than the generated crack length of 140 mm—were inserted into the generated cracks, concrete was poured to create two side walls of 20 mm each.

The CIPs were removed after curing for 90 h, and the total number of days for the production of the specimen was 21. Figure 4d shows the specimen with the mold removed.

**Figure 5:**
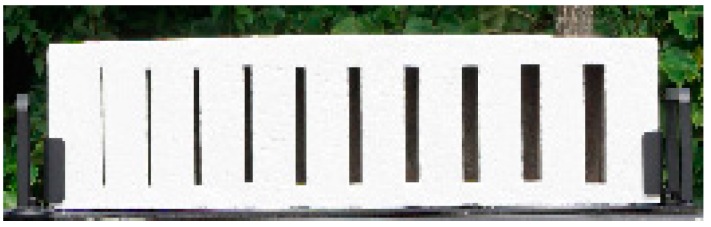
Complete specimen.

As shown in Figure 5, white paint was applied to the specimen, which was installed in a frame to which a handle for the transport of the specimen and a rubber pad were attached. The specimen was produced similarly to the bar target to facilitate the FWHM analysis. However, the spacing between the cracks is 40 mm, which is different from that of the bar target. The produced specimen was 570 mm × 140 mm × 130 mm and weighed about 15 kg.

### 3.2. Crack Specimen Image Acquisition

**Figure 6:**
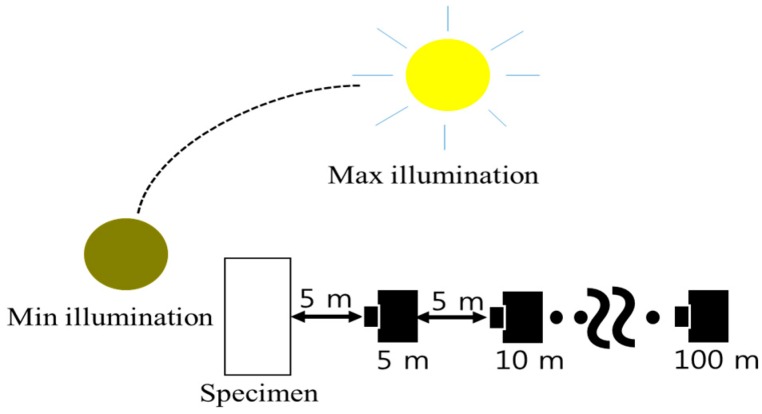
Schematic diagram of crack image acquisition experiment.

Figure 6 shows the experimentation method. A railway track with a lineal distance of more than 100 m was selected as the test site. Image acquisition points from 5 m to 100 m in a straight line from the specimen were marked on the ground at 5 m intervals. For each specific illumination attained depending on the position of the sun, the specimen image could be obtained at the predetermined image acquisition point.

**Figure 7:**
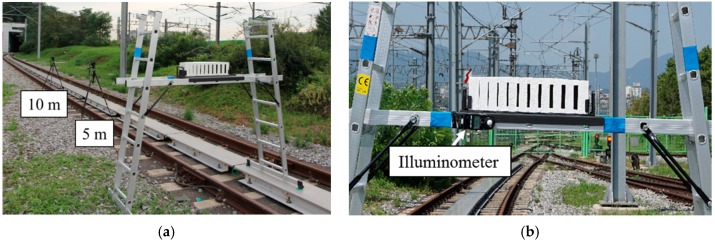
Image acquisition apparatus configuration: (**a**) Camera settings; (**b**) Illuminometer installation.

Figure 7 shows photos of the experimental apparatus for the crack specimen image acquisition. The specimen was mounted horizontally on the specimen holder placed on the railway track, and the illuminometer was attached to the front of the specimen holder. Changes in illumination within a predetermined range depending on the position of the sun were measured using the illuminometer, and the crack specimen images were obtained while moving the camera to the image acquisition points marked on the ground. In order to minimize the changes in illumination during the experiment, an image was obtained using two cameras in less than three minutes, as shown in Figure 7a, and the average value of the illumination measured during the experiment was used as the illumination of the experiment. It was difficult to control the light angle in the outdoor environment. The experiment to find the relationship between crack brightness and lighting angle was thus conducted indoors and the result is shown in Appendix B.

Table 1 shows a summary of the image acquisition conditions of the crack specimen used in the experiment. Of the acquired images, the image with the lowest illumination right before sunset (13 lx) and the image with the highest illumination (52,000 lx) were used in the data analysis. The illuminance according to altitude is shown in Appendix A.

The specifications of the image acquisition camera (ILCE-6000/B KR2, SONY: Tokyo, Japan), lens (SEL35F18, SONY), and illuminometer (T-10A, Minolta: Tokyo, Japan) are summarized in Table 2.

## 4. Analysis of Experimental Results

Table 3 shows a summary of the crack specimen images acquired by the experiment. It illustrates a comparison of the crack specimen images acquired by changing the shooting distance from 5 to 100 m at 13 lx and 52,000 lx with the same specimens.

**Figure 8:**
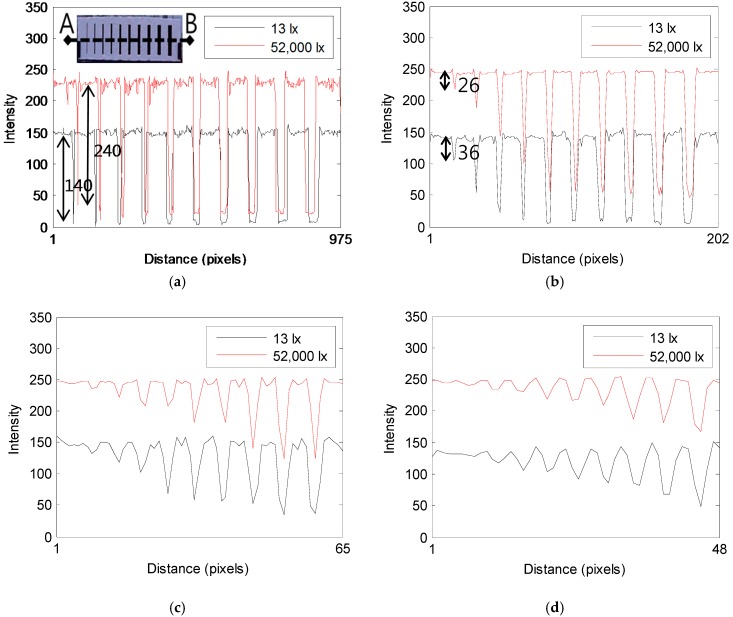
Comparison of the intensity profile between specimen images acquired with the illuminations of 13 lx and 52,000 lx at object distances of: (**a**) 5 m, (**b**) 25 m, (**c**) 75 m, (**d**) 100 m.

A comparison of the images acquired at 13 lx and 52,000 lx reveals that the image acquired at 13 lx exhibits the whole dark contrast, whereas the image acquired at 52,000 lx shows a bright contrast. The images acquired from the distance of 5 m show the contrast of the cracks that are darker than background, which makes it easy to identify the cracks due to the clear contrast. It can be confirmed that as the image acquisition distance increases, the contrast of the cracks becomes blurred and it is difficult to recognize the cracks with small widths located in the left part of the crack specimen in the image acquired from a distance of 100 m. The degree to which the contrast of the crack is blurred as the shooting distance increases is slightly different depending on the illumination of 13 lx and 52,000 lx, and thus there is also a difference in the crack width that can be recognized. In order to analyze the difference in the crack widths that remain visible in each image, the brightness of the image was represented using an intensity profile with a range of 0–255. Figure 8 shows the intensity profile that represents the contrast of the crack specimen image shown in Table 3.

As shown in the upper left corner of Figure 8a, the intensity profile displays the contrast of the dotted area (A–B) in a graph. The place where the contrast becomes lower represents the crack area in the image, and the background exhibits a relatively high value. A comparison of the images acquired at 13 lx and 52,000 lx can confirm that as the contrast of the background has a range of about 150 and 240 at 13 lx and 52,000 lx in the image taken from the distance of 5 m, the difference in brightness appears large, whereas the contrast of the crack shows a range of 7 and 19 at 13 lx and 52,000 lx, indicating that there is no big difference. An arrow of Figure 8a shows the intensity difference between the crack and the background numerically. At 13 lx, the intensity difference was about 140, and there was an intensity difference of 220 at 52,000 lx, showing that the intensity difference between the crack and the background is larger at 52,000 lx than at 13 lx. However, the intensity profile showed a change in the image of Figure 8b, which was acquired by increasing the image acquisition distance to 25 m. The contrast of the crack was shown to rise sharply in the part where the crack width is small on the left area of the graph. As the contrast of the 2 mm crack exhibits 105 and 218 at 13 lx and 52,000 lx, respectively, a rise in the contrast of 98 and 200 can be confirmed. In particular, the contrast rose sharply at 52,000 lx, and the intensity difference from the background due to the rise in the contrast was sharply reduced to 26. The reduction in the intensity difference can be explained as the cause for the 2 mm crack appearing blurred in the image taken from the shooting distance of 25 m in Figure 8c. As the image acquisition distance increased, the contrast of the crack rose sharply, and thus the intensity difference decreased. Therefore, it can be confirmed that it becomes difficult to recognize the crack in the crack specimen image.

### 4.1. Crack Edge Response Analysis Results

**Figure 9:**
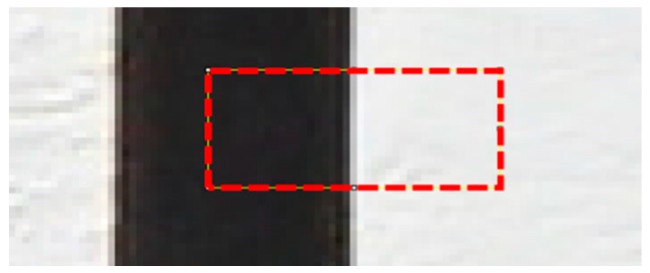
Crack specimen image near the boundary line of the crack to analyze the visibility resolution.

Figure 9 shows a photo in which the crack area is magnified in the crack specimen image. The part shown in red represents the boundary line of the crack and background to extract the edge spread function (ESF). On the basis of the extracted ESF, a line spread function (LSF) was obtained using Equation (3), and then the full width at half maximum of LSF was obtained to analyze the sharpness.

**Figure 10:**
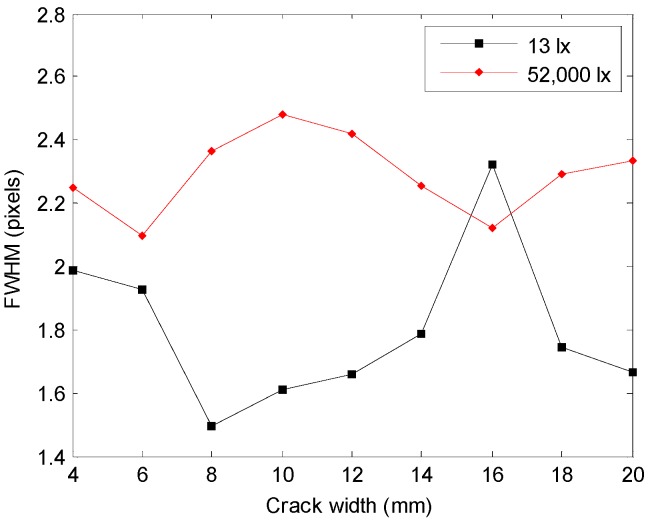
Full width at half maximum (FWHM) of point spread function (PSF) acquired at the boundary line of the crack versus the crack width of the specimen.

In Figure 10, FWHM of LSF at the boundary line of the crack specimen image acquired at 13 lx and 52,000 lx was calculated and illustrated in the graph.

FWHM showed smaller value at 13 lx than at 52,000 lx, and the overall sharpness was found to be better. The average FWHM at 13 lx and 52,000 lx was 1.79 pixels and 2.29 pixels, showing a difference of about 0.5 pixels, and the maximum FWHM difference was 0.87 pixels. Except for the case of the 10 mm crack, the difference in illumination turned out to generate a difference of 8%–36% in the sharpness of the crack boundary line.

### 4.2. MTF Analysis Results

MTF analysis was performed using the edge method and the sine wave method, which are IEC standards. Since the form of the crack specimen is similar to the bar target, two types of analyses could be performed. The edge method that can analyze MTF in a single boundary line without the bar target was used in the short distance image, and the sine wave method was used in the long distance image where the resolution becomes low, and the number of samplings is reduced. First, the edge method was used in the analysis, and the spatial resolution according to the illumination was evaluated by measuring the values of MTF 10% that represents visibility and MTF 50% that represents sharpness in the measured MTF curve.

**Figure 11:**
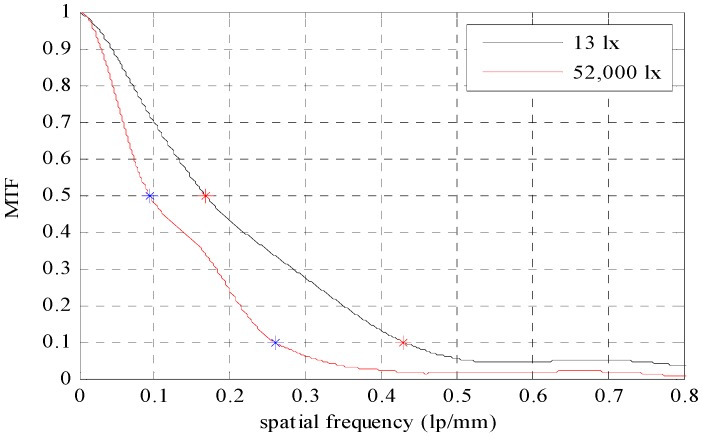
Response of the modulation transfer function (MTF) acquired at the boundary line of the crack image at the 5m object distance using edge method.

Figure 11 shows the response of the modulation transfer function (MTF) acquired at the 12 mm crack boundary line from the 5m image acquisition distance using an edge method.

It was found that the MTF of the crack image acquired at 52,000 lx was more sharply reduced than that of the image acquired at 13 lx. MTF10 of 13 lx and 52,000 lx were 0.430 lp/mm and 0.295 lp/mm, respectively, and the visibility was about 1.6 times higher at 13 lx. MTF50 was 0.167 lp/mm and 0.93 lp/mm, respectively at 13 lx and 52,000 lx, and a sharpness 1.7 times higher was shown at 13 lx.

**Figure 12:**
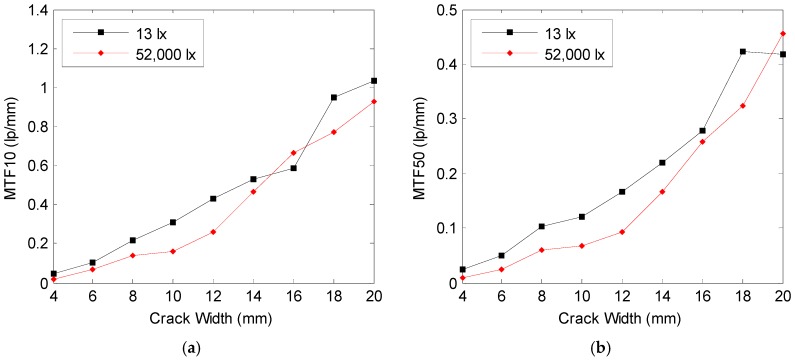
Comparison of (**a**) visibility shown in MTF10, and (**b**) sharpness shown in MTF50 between crack images at the 5 m object distance with the illumination of 13 lx and 52,000 lx.

In Figure 12, MTF10 and MTF50 according to the crack widths of the images acquired at the 5 m image acquisition distance were obtained, respectively, and illustrated in the graph.

A comparison of MTF10 of the images acquired at 13 lx and 52,000 lx reveals that a higher value of the average of 0.080 lp/mm was shown at 13 lx than at 52,000 lx, and better visibility was found at 13 lx. In addition, sharpness showed a tendency to be 0.038 lp/mm higher on average at 13 lx than at 52,000 lx. Sharpness and visibility were increased proportionally with the increases in the crack widths, which is considered to be one of the causes of easier recognition of the area with a large crack width in the crack specimen image.

**Figure 13:**
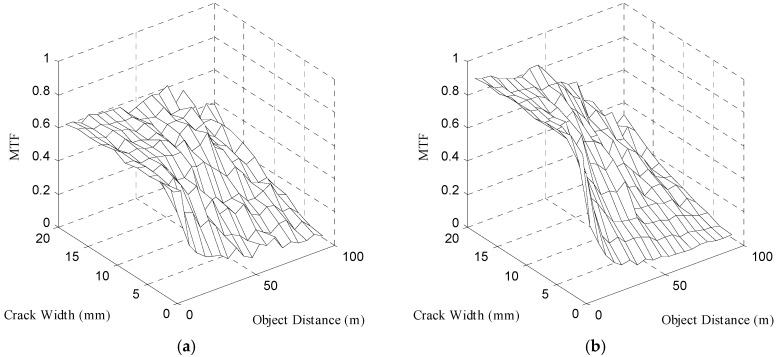
Two-dimensional representation of MTF acquired at the boundary line of the crack image using the sine wave method with the illumination: (**a**) 13 lx, (**b**) 52,000 lx.

Figure 13 shows a graph of MTF analyzed using the sine wave method.

If the image acquisition distance increases, MTF decreases from the crack with a small width due to a reduction in spatial resolution. It can be confirmed that as the image acquisition distance increases, the MTF of the image acquired at 52,000 lx shown in Figure 13b is more rapidly reduced than that of the image acquired at 13 lx shown in Figure 13a. Mraz calculated the minimum recognizable crack width by using MTF10, which corresponds to the spatial resolution of the camera [15]. Therefore, this study analyzed the effects of illumination on the recognizable crack width according to the image acquisition. distance by assuming the crack width that corresponds to MTF10 in the MTF curve obtained experimentally in Figure 13 as the minimum recognizable crack width.

**Figure 14:**
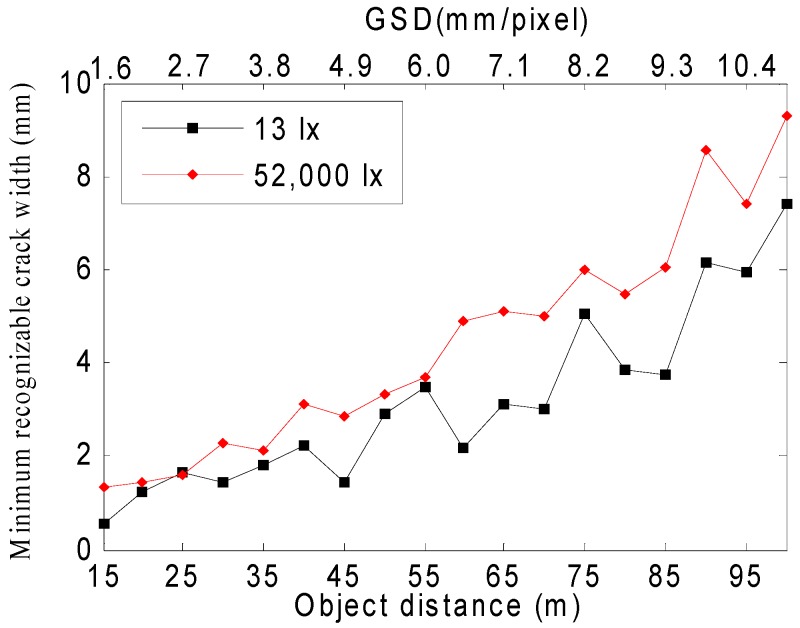
A graph illustrated by calculating the minimum crack width that can be recognized according to the image acquisition distance by assuming MTF10 of the MTF curve as the minimum recognizable crack width.

Figure 14 shows the minimum recognizable crack width calculated using MTF10. As the image acquisition distance increased, the minimum recognizable crack width increased, and a difference in the minimum recognizable crack width occurred depending on the illumination. The minimum crack widths at the maximum image acquisition distance of 100 m were 7.4 mm and 9.3 mm, respectively, at 13 lx and 52,000 lx, showing a 1.9 mm difference. The average minimum recognizable crack width difference depending on the illumination was 1.24 mm, and it was confirmed that a crack of a smaller width could be recognized at 13 lx. The shooting distance for crack recognition can be changed by the camera angle, and thus the ground spatial resolution (GSD) unit (mm/pixel) was added to the horizontal axis in Figure 14. The relationship between spatial resolution and camera direction is shown in Appendix C.

### 4.3. Contrast Sensitivity Analysis Results

**Figure 15:**
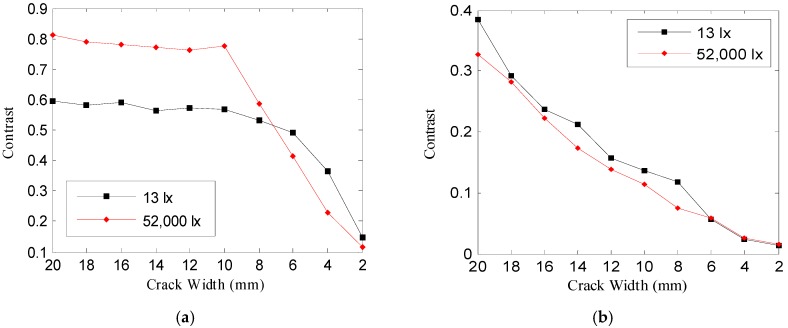
Comparison of contrast of crack images acquired at 13 lx and 52,000 lx, Image acquisition distance: (**a**) 25 m, (**b**) 100 m.

The minimum recognizable crack width obtained using MTF is a value calculated in consideration of the resolution of a camera and has a difference from the sharpness that is visually felt by humans. Figure 15 illustrates the difference between the minimum contrast of the crack and the maximum contrast of the background as the contrast in the acquired crack image.

In the crack image acquired at the 25 m image acquisition distance of Figure 15a, the contrast appeared larger at 52,000 lx than at 13 lx in the case of the crack width of more than 8 mm, and the opposite results were obtained with a crack width of less than 6 mm. These results are the same as in the case where the intensity profile of the crack image acquired at 52,000 lx of Figure 8b rapidly rises as the crack width increases. In the crack image acquired at the 100 m image acquisition distance of Figure 15b, there was almost no difference between the contrast at 13 lx and at 52,000 lx.

**Figure 16:**
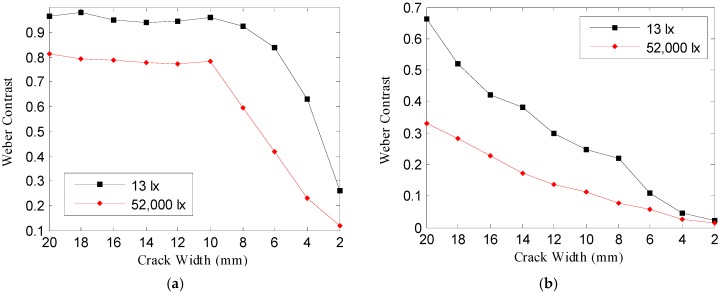
Comparison of Weber contrast of crack images acquired at 13 lx and 52,000 lx, Shooting distance: (**a**) 25 m, (**b**) 100 m.

**Figure 17:**
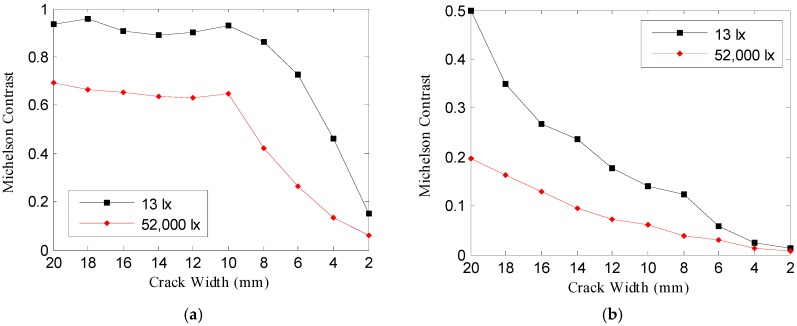
Comparison of Michelson contrast of crack images acquired at 13 lx and 52,000 lx, Shooting distance: (**a**) 25 m, (**b**) 100 m.

However, it can be confirmed that in the image acquired at the image acquisition distance of 100 m in Table 3, there is a difference that the crack shows at 13 lx and 52,000 lx. In order to analyze this difference, which is visually felt, the Weber contrast and Michelson contrast were calculated. Figure 16 and Figure 17 illustrate the Weber contrast (C_W_) and Michelson contrast (C_M_), calculated using Equations (13) and (14).

In the crack image acquired at the 25 m image acquisition distance of Figure 16a and Figure 17a, Weber contrast and Michelson contrast were higher at 13 lx than at 52,000 lx; even a crack of a small width has a great effect on the minimum recognizable crack width—Weber contrast and Michelson contrast were higher at 13 lx. In the crack image acquired at the image acquisition distance of 100 m, there was almost no difference between the contrast at 13 lx and 52,000 lx, as shown in Figure 15; however, as Figure 16b and Figure 17b show, the Weber contrast and Michelson contrast showed higher values at 13 lx than at 52,000 lx in the same way as in the above result. This suggests that there are differences in the degree to which the crack looks blurred in the crack image acquired at the image acquisition distance of 100 m, as shown in Table 3.

**Figure 18:**
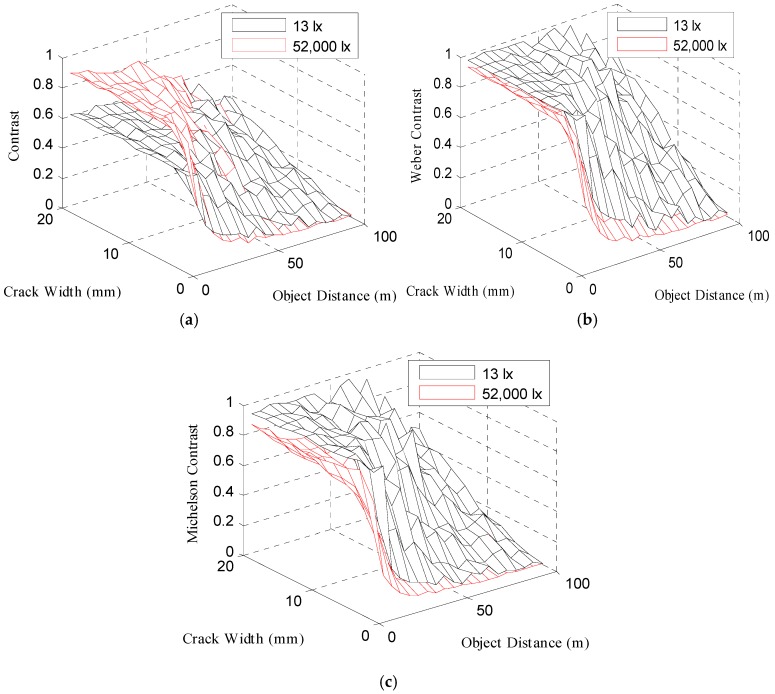
Three-dimensional graphs showing the (**a**) contrast, (**b**) Weber contrast, and (**c**) Michelson contrast changes depending on the image acquisition distance and crack width.

Figure 18 illustrates the contrast, Weber contrast, and Michelson contrast changes depending on the image acquisition distance and crack width in three-dimensional graphs.

When the image acquisition distance increased, the contrast was reduced from the crack with a small crack width. Figure 18b,c shows that the Weber contrast and Michelson contrast exhibited overall higher values at 13 lx than at 52,000 lx.

**Figure 19:**
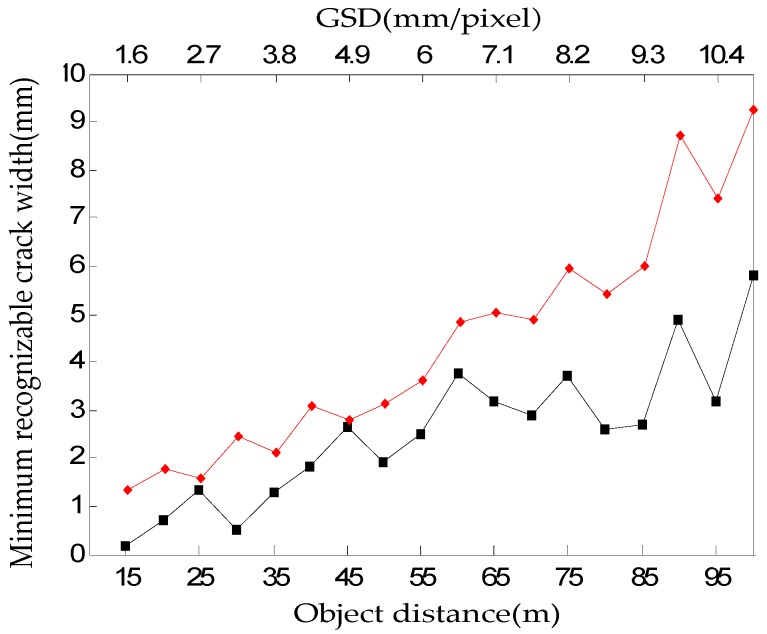
Graph illustrated by calculating the minimum recognizable crack width depending on the image acquisition distance under the assumption that the case where Weber contrast becomes 0.1 is the minimum recognizable crack width.

The Weber fraction for distinguishing the contrast is known to be about 0.1–0.2 [21,22]. In this study, the crack width where Weber contrast becomes 0.1 in Figure 18b was assumed to be the minimum recognizable crack width, and then the minimum recognizable crack width was estimated depending on the image acquisition distance. Figure 19 shows two cases of the minimum crack widths depending on the image acquisition distance at 13 lx and 52,000 lx.

**Figure 20:**
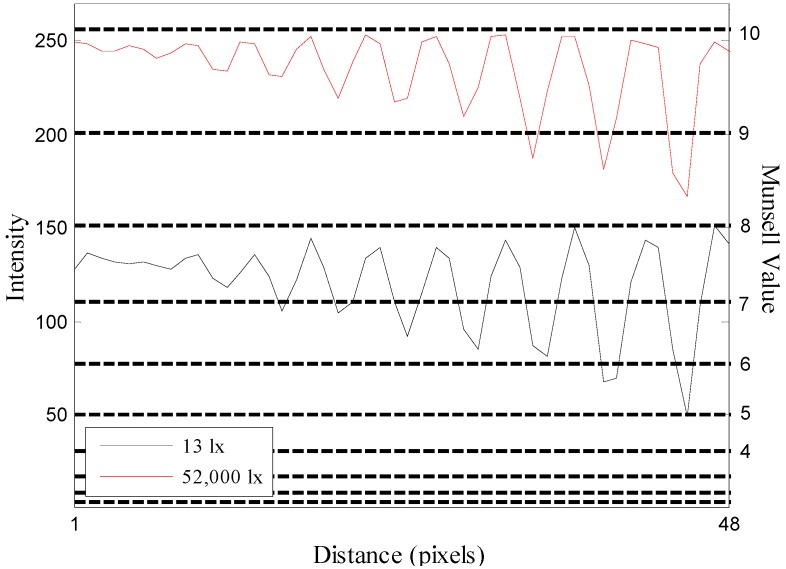
Intensity profile and Munsell value of crack images acquired at the image acquisition distance of 100 m.

The average of the minimum recognizable crack width of the crack image acquired at 13 lx and 52,000 lx was 2.53 mm and 4.40 mm, showing a difference of 1.87 mm. The minimum crack width of the crack image acquired at the maximum crack image acquisition distance of 100 m was 5.81 mm and 9.34 mm, respectively, at 13 lx and 52,000 lx, showing a difference of 3.44 mm. Thus, it can be confirmed that a crack of a smaller width can be recognized in the crack image acquired at 13 lx. In addition, the Munsell value was used to analyze the effects of illumination on crack recognition in terms of visual perception. Figure 20 illustrates the intensity profile (Figure 8d) of the crack images acquired at the image acquisition distance of 100 m, along with the Munsell value.

The Munsell value shows perceptual characteristics, illustrating that as the brightness is higher, the change of brightness that can be perceived decreases. The difference of Munsell value between the background and the crack image taken at 52,000 lx becomes smaller than that of the image taken at 13 lx. As for the Munsell value of the crack image acquired at 52,000 lx, the background is about 10, and the crack wider than 16 mm is 9, and therefore the brightness can be distinguished by the value difference of 1. However, since the cracks smaller than 14 mm show a value difference of less than 1 from the background, it is difficult to distinguish the difference of brightness between the crack and the background. On the other hand, as the value of the crack image acquired at 13 lx is 8 in the background, showing a value difference of more than 1 from the cracks wider than 8 mm, the brightness difference can be distinguished up to the crack of a smaller width. As a result, the analysis found that the crack widths that show a value difference of more than 1 from the background were 8 mm and 14 mm at 13 lx and 52,000 lx, and therefore the minimum crack width that can be recognized at 13 lx was smaller.

## 5. Conclusions

This study objectively evaluated the effects of crack image acquisition conditions for concrete structures on the recognition of the cracks in crack images through outdoor experiments and closely examined the ranges of crack width that can be recognized depending on the illumination. Through the experiments, a specimen with a crack that has a certain width was produced similarly to the bar target. Image analysis techniques such as MTF and contrast sensitivity were used, and the crack widths that can be recognized in terms of visual perception were analyzed. MTF analysis results showed that MTF10 and MTF50 were found to be smallest in the crack images taken at 52,000 lx, which is the maximum illumination during the daytime, and as the shooting distance increased, the recognizable crack widths became relatively large, which makes it more difficult to recognize the cracks. The analysis on the sharpness in the boundary line of the cracks found that the sharpness of the crack image taken at 13 lx was higher than that of the crack taken at 52,000 lx, but the difference was not significant. The changes of the contrast sensitivity depending on the shooting conditions were investigated based on the analysis on the Weber contrast and Michelson contrast of the crack images. The analysis results confirmed that the overall Weber contrast and Michelson contrast were lower in the crack images taken at 52,000 lx, compared to the crack images taken at 13 lx, and the crack image taken at 52,000 lx in the daytime environment has a larger effect on the sharpness reduction than the crack image taken at 13 lx in the low-illumination environment.

If the illuminance is high or low, the contrast of the crack and concrete background increases or decreases. In this study, the maximum illuminance of 52,000 lx and the minimum illuminance of 12 lx were selected as experimental values in order to analyze the effects of the contrast changes on the crack recognition. If illuminance is higher or lower than that used in the experiment, the contrast increases or decreases. Even in this case, the effects can be estimated with reference to this paper.

The contrast and sharpness of the crack are among the most important variables even when cracks are extracted using visual perception as well as image processing techniques, such as thresholding or boundary extraction. Therefore, it is expected that the analysis results of MTF and contrast sensitivity can be used as quantitative indicators that represent the possibility of detecting cracks.

## Figures and Tables

**Table 1:** Environmental conditions for crack specimen image acquisition experiment.

Image Acquisition Distance (m)	5~100 (Step 5)	
Acquisition time	19:08~19:11	12:54~12:57
Illumination (lx)	7~18	52,000~52,000
Azimuth	N281°00′16″	N191°09′10″
Altitude	−03°11′56″	059°04′40″

**Table 2:** Detailed specifications of the apparatuses used in the experiment.

Apparatuses	Items	Specification
Camera	Resolution	6000 × 4000
	Sensor type(size)	APS-C (23.2 × 15.4 mm)
Lens	Focal length	35 mm
	Maximum Aperture Range	F1.8
	Min Aperture	22
Illuminometer	Receptor	Silicon photocell
	Measuring range	0.01 to 299,900 lx; 0.001 to 29,990 fcd

**Table 3:** Crack specimen images acquired by varying the image acquisition distance at 13 lx and 52,000 lx illuminations.

	Image Acquisition Distance (m)			
	5	25	75	100
13 lx	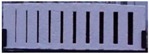	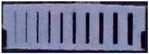	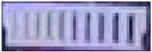	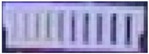
52,000 lx	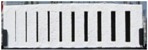	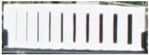	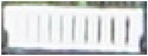	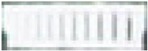

**Figure B1:**
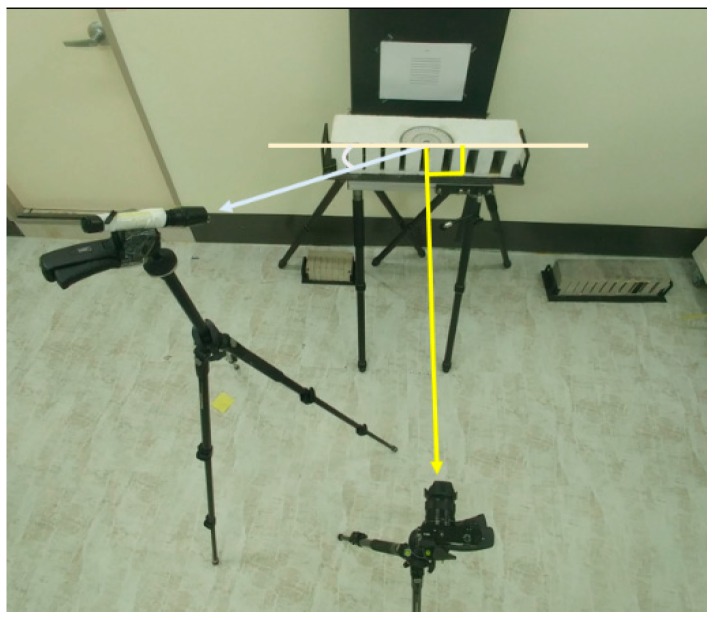
Test setup for analysis of lighting angle and intensity of cracks.

**Figure B2:**
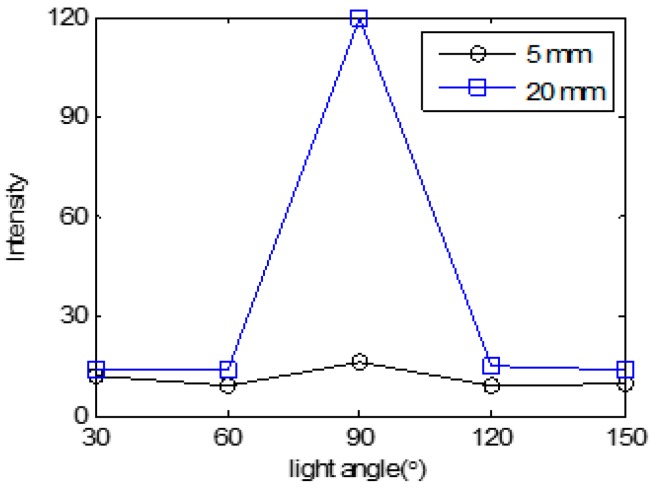
Variation in brightness of crack depending on light angle and crack width.

## Appendix A. Daylight Illuminance

The illuminance may vary depending on the region, and therefore the illuminance value should be estimated according to the altitude of the sun. Lee represented the horizontal illuminance (L_H_) according to the altitude (α) as in Equation (A1) [23]. Perez-Buros (2010) [24] measured vertical illuminance according to the azimuth and represented the relationship between horizontal illuminance (*L_H_*) and vertical illuminance (*L_V_*) using Equation (A2) as shown below:

(A1) $$L_{H}=-6710.52+2776.24\alpha -11.6234\alpha^{2}$$

(A2) $$\frac{L_{v}}{L_{H}}=0.109\sin{\left(\alpha \right)}^{-0.6777}$$

The azimuth and altitude of the sun during the experiment are shown in Table 1. Since the front of the specimen heads towards the azimuth of 159°, the vertical illuminance in a south direction was measured. Horizontal illuminance represents the illuminance on the pavement, and the vertical illuminance can represent the one that illuminates the walls.

## Appendix B. Relationship between Crack Brightness and Lighting Direction

MTF and contrast sensitivity are calculated using the crack and background brightness. Since this was conducted as outdoor test using the sunlight as light source, it’s difficult to consider the effect on MTF and contrast sensitivity depending on lighting direction. Therefore, the effect of lighting direction on the analysis was reviewed using indoor lighting.

In tests, a camera was set in front of the crack and the image was photographed while the light angle was changed by 30° intervals from 30° to 150° (Figure B1). Then the variation of brightness of the crack and background in the image was analyzed so as to identify the potential effect of light angle on definition and contrast sensitivity. Table B1 shows a summary of the crack specimen images acquired by the experiment.

**Table B1:** Changes in brightness in crack depending on light angle and crack width.

		Light Angle (θ)				
		30	60	90	120	150
Crack width (mm)	5					
	25					

Figure B2 shows the intensity changes of cracks. A crack has geometric features with extended depth for the width and the darkness of the crack is attributable to low incident light on the inner side of the crack due to the geometric shape of the crack. For such a reason, the crack looks dark when there is lighting from the side. When the light is perpendicular (90°) to the crack, the amount of light reaching the inner side of crack is highest. In such a case, the brightness varies depending on the crack width. In the case of a narrow width (5 mm), the light is reflected into the inner side despite the light being at the right angle and thus the brightness is low, indicating 16 only, though it rose to slightly higher than that at oblique angles. However, in the case of a wide width (25 mm), relatively more incident light becomes available, which raises the brightness up to 120. If the crack brightness increases, the contrast with the background decreases, and thus the contrast sensitivity and MTF decrease.

However, it may be assumed, in fact, that most cracks have an extended depth, and sunlight being perpendicular to the inner side of the crack is very rare. In the case of side lighting, low brightness was maintained irrespective of the angle, and the test conducted in this study considered the illumination intensity alone.

## Appendix C. Relationship between Spatial Resolution and Camera Direction

When the camera angle was changed, the distance between the crack and camera increased accordingly; transverse spatial resolution also increased, as indicated in Equation (C1), and longitudinal spatial resolution increased depending on cos*θ*, as shown in Equation (C2), which indicates that the crack width that is measurable at the same distance is reduced [25].

(C1) $$R_{t}=\frac{S_{\mathrm{pixel}}\times \sqrt{h^{2}+l^{2}}}{f}$$

(C2) $$R_{l}=\frac{S_{\mathrm{pixel}}\times \sqrt{h^{2}+l^{2}}}{f\times \cos\left(pitch\right)}$$

where *R_t_* is the transverse spatial resolution, *R_l_* is the longitudinal spatial resolution, *H* is the height of the tower, *l* is the ground distance, *pitch* is angle between horizontal plane and camera direction, *S_pixel_* is the pixel size of the camera, and *f* is the focal length.
